# BiowareCFP: An Application-Agnostic Modular Reconfigurable Cyber-Fluidic Platform

**DOI:** 10.3390/mi13020249

**Published:** 2022-02-02

**Authors:** Georgi Tanev, Winnie E. Svendsen, Jan Madsen

**Affiliations:** 1 DTU Compute, Technical University of Denmark, 2800 Kongens Lyngby, Denmark; geta@dtu.dk; 2 DTU Bioengineering, Technical University of Denmark, 2800 Kongens Lyngby, Denmark; wisv@dtu.dk

**Keywords:** digital-microfluidics, cyber-fluidic systems, application agnostic, platform-based design

## Abstract

Microfluidic biochips have been in the scientific spotlight for over two decades, and although technologically advanced, they still struggle to deliver on the promise for ubiquitous miniaturization and automation for the biomedical sector. One of the most significant challenges hindering the technology transfer is the lack of standardization and the resulting absence of a common infrastructure. Moreover, microfluidics is an interdisciplinary field, but research is often carried out in a cross-disciplinary manner, focused on technology and component level development rather than on a complete future-proof system. This paper aims to raise awareness and facilitate the next evolutionary step for microfluidic biochips: to establish a holistic application-agnostic common microfluidic architecture that allows for gracefully handling changing functional and operational requirements. Allowing a microfluidic biochip to become an integrated part of a highly reconfigurable cyber-fluidic system that adopts the programming and operation model of modern computing will bring unmatched degrees of programmability and design reusability into the microfluidics field. We propose a three-tier architecture consisting of fluidic, instrumentation, and virtual systems that allows separation of concerns and promotes modularity. We also present BiowareCFP as a platform-based implementation of the outlined concepts. The proposed cyber-fluidic architecture and the BiowareCFP facilitate the integration between the virtual and the fluidic domains and pave the way for seamless integration between the cyber-fluidic and biological systems.

## 1. Introduction

The miniaturization and integration in the microelectronics industry have been exponentially growing since the introduction of the first commercially available microprocessors in the early 1970s. The commercial debut of the microprocessor marked the beginning of a new era, where computers became compact and economically feasible for use in everyday life. Fast forward 50 years, and we see a ubiquitous mixture of personal computers, portable devices, and various cyber-physical systems connecting our tangible world with the emerging virtual world.

In a similar manner, the miniaturization and integration of the traditional wet laboratory processes started gaining traction in the early 1990s when the first ideas about miniaturized total chemical analysis systems (µTAS) were introduced [1]. Active microfluidics allowed for the control of molecular concentrations in fluids over space and time, and they became an underlying technology for lab-on-a-chip (LoC) applications. The LoC devices are often referred to as microfluidic biochips due to their compactness and ability to programmatically deal with biological species.

The vision for microfluidic biochips overtaking the field of medical and biological analysis has been described as the motivation for countless research papers. Lower reagent volumes, automation potential, shorter hands-on time, miniaturization, scalability, and reduced overall costs are often articulated as key selling of the microfluidic technology. However, despite the strong technological development and great potential, 30 years later, the microfluidic biochips still mostly remain in the academic domain, where scientists focus on technological advancements and writing scientific papers about them [2].

The field of microelectronics was first driven forward by engineering and academic interest, but it was profoundly supported by the revenue generated from the steadily increasing market demanding higher-performance computers. Likewise, finding a commercially viable “killer application” that will fuel the technological transfer and overcome the remaining technological challenges is, in theory, the key to pushing microfluidic biochips past the proof-of-concept stage.

Some of the most exciting microfluidic applications relate to the healthcare sector or the emerging biological computing and synthetic biology fields. Unfortunately, the research record shows that seeking a commercial “killer application” (if focusing on commercialization at all) often appears in the context of short-term research goals. This strategy inherently lacks the means to facilitate sustainable technological development; i.e., it produces proof-of-concept prototypes that have limited applicability to a small subset of problems. Without a common infrastructure, a significant portion of each research endeavor often goes into trivial but cumbersome microfluidic engineering, establishing fabrication processes, and building instrumentation systems, instead of focusing on what is important, i.e., bringing value and solving real problems.

In contrast, the microelectronics and computer industries heavily rely on standardization and design reuse, where a new system is often primarily made of already existing modules. It is seldom the case that a computer is built from scratch or software is written without an external codebase and extensive toolchain. Modern computer systems can be reconfigured and reprogrammed, which allows focusing on what is essential—building applications and solving complex problems quicker, better, and at a reasonable cost.

Nevertheless, one might ask why the parallels between the computer and microfluidic biochips are relevant. The intersection between the cyber and the physical domains is what we believe is immediately relevant to the microfluidic biochips, i.e., a programmable cyber-fluidic architecture that links the computational capabilities of the virtual world with the liquid and biological handling capacity of the microfluidic biochips. Allowing a microfluidic biochip to become an integrated part of a highly reconfigurable cyber-fluidic system that adopts the programming and operation model of modern computing holds the potential to disrupt the fields of life and computer science. Undoublty, the underlying biological mechanisms are far more involved than the computer system, but the fluidic handling in its essence is not much different from operations performed with virtual data.

**Figure 1:**
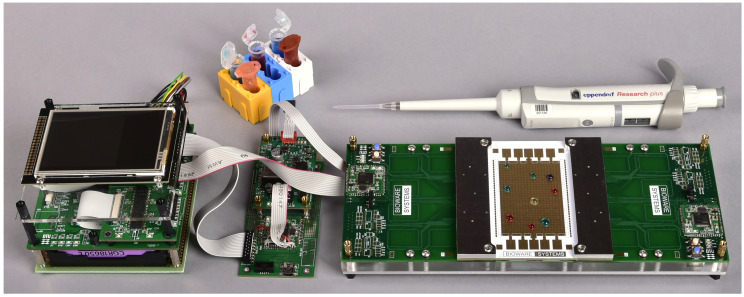
The BiowareCFP instrumentation system instance.

The rest of this paper focuses on what we believe is the missing link for establishing a common microfluidic infrastructure—a cyber-fluidic architecture that has the potential to mark the next evolutional step of the microfluidics development. We argue about the advantages and demonstrate the feasibility of the suggested architecture by unfolding the concepts into a modular cyber-fluidic platform instance called BiowareCFP. The platform prototype is shown in Figure 1. The design is governed by a holistic, interdisciplinary approach striving to create a system that can adapt to changing functional and operational requirements throughout its lifecycle. In this paper, we argue about choosing the fluidic handling technology and discuss the various architectural, building, and operational characteristics of the envisioned full-stack cyber-fluidic platform. With this endeavor, we are not attempting to find the “killer application.” We are instead proposing a cyber-fluidic architecture that has the resources to aid and accelerate the research process by bringing unmatched amounts of design reusability and programmability into the microfluidics field.

### 1.1. The Importance of the Fluidic Handling Technology

**Figure 2:**
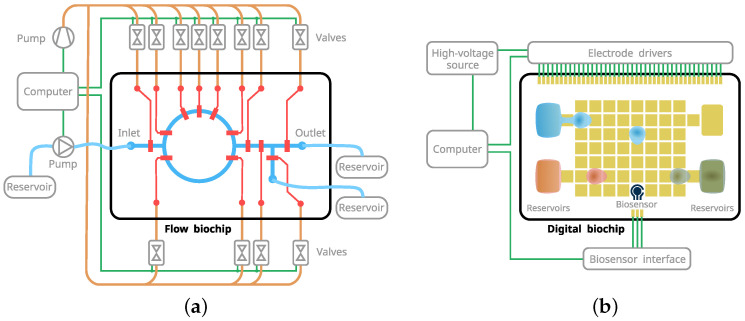
Microfluidic biochip examples with required external instrumentation. (**a**) Channel-based continuous flow biochip. The on-chip valves are controlled trough off-chip pressure valves and pumps. (**b**) Digital biochip with integrated biosensor.

There are two dominant active microfluidic handling technologies: channel-based continuous flow and planar digital microfluidics, as shown in Figure 2. As the name implies, the channel-based systems operate with continuous liquid flow physically confined in a network of fixed channels. The transport mechanism is based on differential pressure pushing the fluidic stream. An external pump creates the pressure, and a valve control network can be used to govern the flow. The channel-based microfluidics often has constrained programmability, although attempts to achieve a general programmable architecture have been presented [3]. The limitations are posed by the fixed one-way channel structure, the external and often involved valve control, and the inherent difficulties with discrete volume management.

In contrast, digital microfluidics allows the manipulation of individual droplets on a planar surface. Droplet actuation is achieved by an electrical field, thereby allowing for a relatively simple first-order digital-to-fluidic interface. Handling discrete volumes provides a straightforward method for controlling chemical species’ concentrations, and it closely resembles the operations typically performed as a part of a benchtop laboratory protocol. The lack of physically defined channels and potentially bulky external instrumentation favors programmability and moderate the scalability challenges.

The two technologies have their strengths and weaknesses. Although we do not advocate that digital microfluidics is technologically or functionally superior, we believe that digital microfluidics, with its simple construction and elegant operation principle, appears to be better suited for creating a universal programmable cyber-fluidic processor capable of handling various chemical species. While the strategies for creating a universal cyber-fluidic system certainly extend beyond the boundaries of the two mentioned microfluidic technologies, we chose digital microfluidics as the rational point of departure.

### 1.2. Digital Microfluidics

Digital microfluidics (DMF) operates on a planar array of individually addressable electrodes coated with a hydrophobic isolation layer. Droplet actuation is achieved by controlling the surface wettability as a function of an electric potential applied to pairs of adjacent electrodes. This technique is known as electrowetting on dielectric (EWOD) [4], and it provides a first-order electrical-to-fluidic control interface. Droplets with various biochemical compositions can be programmatically moved, merged, and split, thereby serving the purpose of discrete fluidic vehicles and miniature reaction chambers. This elegant control method enables multiple reactions to be carried out on the same device without cross-talk between different samples or reagents. Combining the fluidic handling capabilities of DMF with miniaturized analytical methods allows for implementing truly integrated sample-to-answer LoC devices. These devices are often referred to as digital biochips due to their discrete droplet manipulation and their ability to facilitate chemical or biochemical assays.

**Figure 3:**
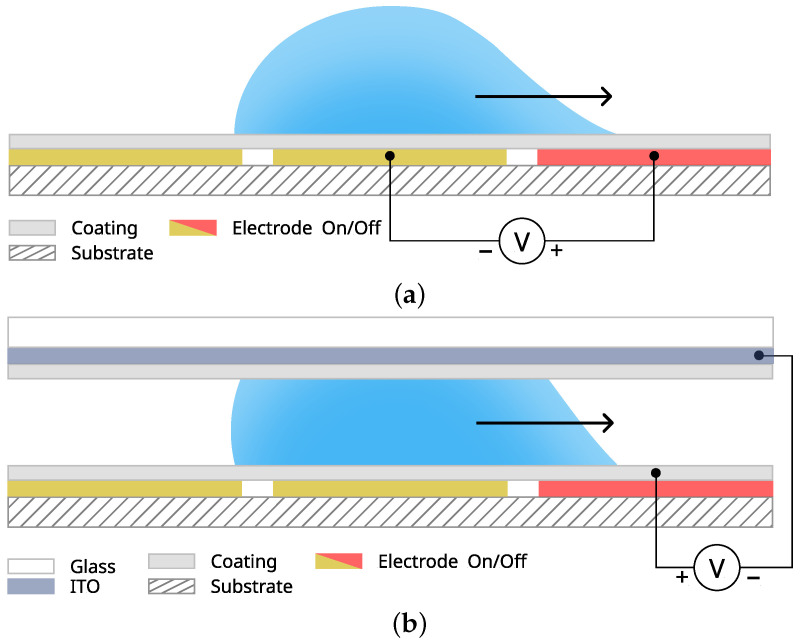
Side-on views of open and closed digital microfluidic droplet actuators. (**a**) Open configuration. (**b**) Closed configuration.

**Figure 4:**
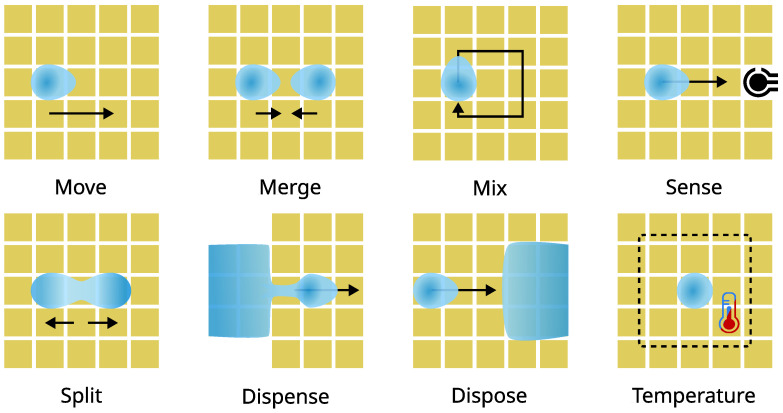
Droplet operations.

Digital biochips exist in open or closed configurations, as shown in Figure 3. The open configuration consists of a single planar substrate patterned with an electrode array. The array must be coated with a hydrophobic insulating layer preventing ohmic current from passing through the actuated liquid sample. Although relatively simple, the open digital biochip configuration supports all fluidic operations shown in Figure 4, but splitting and dispensing appear to be challenging due to the high actuation force required to overcome the surface tension [5]. The closed configuration uses a hydrophobic coated conductive top plate as a ground-referenced electrode. The droplet sample is sandwiched between the electrode substrate and the top plate, allowing precise droplet splitting and dispensing from reservoirs [6]. Due to their construction, the open and closed systems present a range of advantages and limitations. For example, the open system is more straightforward to construct, and it provides direct access for loading and extracting reagents. However, it requires a higher actuation voltage, it is prone to contamination and evaporation, and it does not support controlled droplets split [5]. In contrast, the closed digital biochip configuration provides controllable splitting [7], requires lower actuation voltage, and reduces or mitigates evaporation and contamination, especially when using an immiscible filler fluid [8]. Nevertheless, the enhanced functionality comes at the costs of device fabrication and operational complexity—e.g., adding a top plate and filler fluid complicates the sample loading and unloading processes.

### 1.3. Sensors and Actuators

A digital biochip’s functionality can be further enhanced with electrochemical [9,10,11] and optical [12,13] detection methods. Moreover, actuators, such as a magnetic stage [14] or heaters providing temperature control [15,16], have been demonstrated to add an extra layer of functionality. This integration allows a wide range of benchtop protocols, such as chemical and enzymatic assays, immunoassays, clinical diagnostics, nucleic acid manipulation, and cell-based applications [17] to be adapted, miniaturized, automated, and implemented on an integrated digital biochip.

### 1.4. Technological Challenges

Although the EWOD appears to be a straightforward droplet actuation method, employing it for digital biochips poses a wide range of technological challenges. Device properties such as substrate material, fabrication methods, electrode geometry, coating material, and gap height must be carefully chosen to achieve reliable and repeatable droplet actuation [18]. Moreover, electrode excitation voltage in the range of hundreds of volts is typically needed to produce sufficient droplet propulsion forces. The high actuation voltage requires nontrivial instrumentation electronics consisting of a high-voltage power supply, electrode drivers, mechanical interfacing, and a programmable control system. The instrumentation challenge becomes even more articulated in the context of increasingly more demanding applications [17,19,20], when the device form factor, cost, sensors, and actuators integration are considered. Coupling the fluidic handling of the digital biochips with the instrumentation and control systems creates a unique interdisciplinary paradigm that we recognize as a cyber-fluidic system, i.e., a fluidic handling device instrumented by a network of computer systems.

### 1.5. The Present Approach

Despite two decades of digital microfluidics development [21], a large portion of the research still follows a narrow, linear, and technology or application-specific path. While essential for establishing and developing a new technology, this approach fails to account for the breadth and current maturity level of the field. The digital microfluidics research largely remains cross-disciplinary; i.e., challenges from one discipline are recognized, and assumptions are made from the viewpoint of another discipline. Without attempting a comprehensive classification, most digital microfluidics research falls in one of three key research areas: (i) developing better microfluidic components, (ii) implementing and demonstrating novel bioassay and applications, or (iii) focusing on the instrumentation and automation aspects. The research contributions in these areas usually come from scientists with domain expertise, correspondingly in physics and material science, biology and nanotechnology, or computer science and electrical engineering. The discrepancies between the domains are large, which, combined with the complexity of the cyber-fluidic systems, naturally leads to unrealistic assumptions regarding digital biochip functionality, reliability, usability, scalability, sustainability, cost, and programmability. The convenience of making technological advancements or implementing an application in a cross-disciplinary context is undeniable; however, such developments under questionable assumptions seldom carry the merits to push the complex technology beyond the proof of concept stage. Moreover, incremental developments made outside of a sustainable framework are challenging to reproduce and seldom carry the prospect of being embraced by other researchers. Consequently, although valuable, such technological advancements often get lost.

Designing and building a full-stack cyber-fluidic system is beyond the scope of a single technological domain. A multidisciplinary effort where different disciplines work closely together is considered a minimum requirement. However, full-stack cyber-fluidic development greatly benefits from an interdisciplinary approach where knowledge and methods from the different technological domains are blended to solve the challenge at hand. Although one can argue that this reasoning seems obvious, it raises the question of why the field of cyber-fluidic systems yet remains somehow immature. There is a lack of standardization on all levels. A common digital biochip form factor is virtually non-existent, and electrode layout is often done in graphical editing software, besides the existence of attempts for layout automation [22,23]. Apart from a few domain-specific languages [3,24] digital biochip programming is performed with custom scripting languages with a lack of flow control constructs.

Nevertheless, several instrumentation systems have emerged as cornerstones on the path towards programmable cyber-fluidic systems. The most notable example is the open-source DropBot system [25], which at the time of its release provided an opportunity to enter into digital microfluidics research without investing hundreds if not thousands of man-hours into developing new custom instruments [26]. Another cornerstone in lowering the entry point was the OpenDrop system [27], which has limited capabilities compared to DropBot, but it is significantly simpler to build and operate. Without attempting completeness, the two systems mentioned above served as the inspiration for several other custom-built digital microfluidic research devices [28,29,30]. There are undoubtedly many other instrumentation prototypes with different levels of integration and technical capabilities. However, their designs often appear to be driven by application-specific research goals, and naturally, they remain in the shadow of the reported microfluidic or application advancements. Although crucial for research and emerging applications, creating countless limited instrumentation devices is hardly efficient in the long run.

### 1.6. The Need for a New Approach

Recognizing the lack of standardization and reflecting on the interdisciplinary paradigm—i.e., the knowledge and methods from the different technological domains are combined to solve problems—allows for drawing parallels between the digital microfluidics domain and the heavily standardized and automated field of computer science and microelectronics [31,32]. We acknowledge the absence of a common fluidic hardware/software architecture as a key factor preventing digital microfluidics from unfolding their potential into a fully programmable cyber-fluidic system. We address this problem by a holistic, interdisciplinary approach and propose a loosely coupled three tire cyber-fluidic architecture consisting of fluidic, instrumentation, and virtual systems. Each of these systems has been purposely devised considering the intrinsic dependencies of the cyber-fluidic domains, resulting in the modular and reconfigurable cyber-fluidic platform presented here.

## 2. Platfrom Architecture

**Figure 5:**
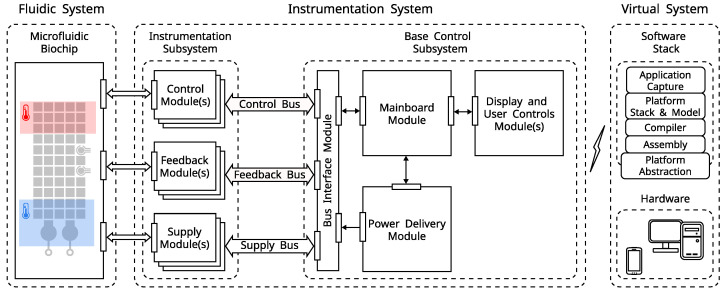
The cyber-fluidic platform architecture. The fluidic system is based on a microfluidic biochip, and the virtual and instrumentation parts represent the cyber part of the system.

A platform-based modular design approach [31,33] allows balancing system complexity and development effort while maintaining functional and performance constraints. This design approach also enables building a common framework for cross-disciplinary research; namely, design assumptions can be verified in practice, and incremental technological advancements become part of the platform’s functionality. The core design goals for the cyber-fluidic platform are the ability to accommodate for changing functional requirements, ease of use, and to implement a flexible programming model. The derived platform architecture, shown in Figure 5, consists of fluidic, instrumentation, and virtual systems. While the fluidic system is regularly in the research spotlight, there are only a handful of instrumentation systems. The most underdeveloped part remains the virtual system, a deficiency often incorrectly justified with the lack of a comprehensive control system due to the relatively simple design and operation of the digital biochips.

### 2.1. Overview

The fluidic system is responsible for the liquid sample handling, and it includes a digital biochip and any associated auxiliary sensors and actuators. The fluidic system is the most delicate and technologically challenging of the three platform parts, and therefore, the modularity and functional mapping of the instrumentation and the virtual systems are governed by the features and limitations of the fluidic system.

The instrumentation system is the link between the cyber and fluidic components of the platform (see Figure 5), and it is designed to provide a reconfigurable and evolvable approach to digital microfluidics instrumentation. The core of the system is a combination of instrumentation modules connected to the mainboard module through a custom bus interface, as shown in Figure 5. The instrumentation needs of the fluidic system were previously generalized into control, feedback, and supply categories [31]. This separation is reflected in the instrumentation system as one-to-one functionality mapping to control, feedback, and supply modules responsible for performing a particular instrumentation task. For instance, an electrode driver module is responsible for driving the electrodes, and a high-voltage power supply module provides the driving voltage. A battery power delivery module and local user control facilitate portable standalone operation.

A full-stack software toolchain, including manual and automated instrumentation system control, high-level protocol capture, translation, and execution on the digital biochip, can be built on top of the cyber-fluidic platform. The software stack represents the virtual system (see Figure 5), and its most important aspect is defining and implementing a standard microfluidic instruction set architecture (μfISA). The μfISA enables abstracting and decoupling the programming model from the actual fluidic and hardware implementation. Moreover, it establishes a clear programming model that promotes modularity and reusability.

### 2.2. Modularity

The modular design approach promotes encapsulation and allows agile development of different functionalities. The platform modules interconnect through defined interfaces on the physical, electrical, and protocol levels. Examples of such interfaces are the control, feedback, and supply communication buses shown in Figure 5. Moreover, a unit size form factor (1 U = 25 mm) for the fluidic and instrumentation systems components is used to secure mechanical compatibility between the different components. The modularity occurs on the virtual level as well, where the different components of the software stack are loosely coupled; i.e., functionality is segmented, and communication between the different software tools is done through established data formats. This approach allows for a service orientated architecture, where, for instance, a protocol capture program can pass an intermediate representation of a programmed protocol to a platform-specific compiler. The underlying mechanism is based on service interfaces that specify the input and output points of each software tool. This allows services to be called with little or no knowledge about the underlying implementation.

### 2.3. Platform Instance

**Figure 6:**
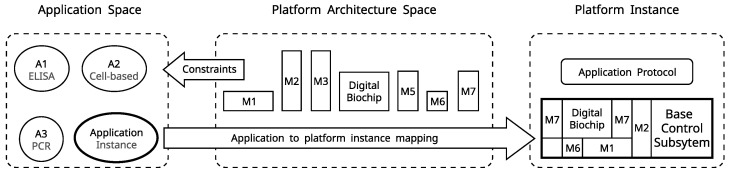
Application space to platform instance mapping. The platform instance is composed of a library of available modules (*M*) to match the fluidic handling needs of a particular application (*A*). Figure inspired from [33].

The modular design and the one-to-one functionality mapping in the instrumentation system allow for creating an application-specific platform instance from a library of building blocks. This process is illustrated in Figure 6, where an application instance from the application space is mapped to the platform architecture space and composes a particular platform instance. The available instrumentation capabilities in the platform function space also pose a constraint to the application space; i.e., a biosensor can not be used if a suitable instrumentation module is not present in the function space. However, the platform function space library can be expanded on an ad hoc basis when the need emerges. Both top-down and bottom-up approaches are possible where an application instance is compiled to a platform instance, or the fluidic system defines the instrumentation needs. The cyber-fluidic platform modules are designed to be plug-and-play; therefore, the platform configuration is automated rather than left to the operator.

### 2.4. Fluidic System

The EWOD droplet actuation relies on the properties and quality of the digital biochip electrode array the hydrophobic insulation layer. This splits the fabrication of the digital biochips into four parts: (i) fabricating the electrode patterned substrate, addressed in Section 2.4.1; (ii) the fabrication of the hydrophobic isolation layer discussed in Section 2.4.2; (iii) the top plate construction discussed in Section 2.4.3; and (iv) any other associated sensors and actuators, as outlined in Section 1.3. The decomposition and modularization of the fluidic system allow for reconfiguration and streamline the development process.

#### 2.4.1. Substrate and Electrode Array

Glass and silicone substrates with electrode patterns created by photolithographic processes are de facto the standard fabrication method for digital biochips. Another alternative includes printing on low-cost substrates, such as paper [9] or plastic [34]. Although capable, these approaches are either cost-prohibitive or difficult to scale to several hundred or even thousands of individually addressable electrodes. Scaling up the number of electrodes is comparable to increasing the memory of a computer system; a larger electrode array provides more droplet storage and allows for more operations to be carried out in parallel.

Fast prototyping printed circuit board (PCB) services have emerged in recent years, and the current fabrication capabilities readily allow for low-cost multilayer PCBs with 100 μm× 100 μm track width and spacing, and interconnects with 200 μm (diameter) holes and 450 μm (diameter) annular rings. The size, the cost of a few dollars per board, and the few days of turn-around time make low-cost PCB fabrication a particularly attractive choice for fabricating the actuation electrode array for digital biochips. A distinct technological advantage is the ability to create two-dimensional arrays of individually addressable electrodes due to the multilayered structure and the interconnects.

#### 2.4.2. Hydrophobic Isolation Coating

The typical coating approach for digital biochips is a two-step process that consists of the deposition of an isolation layer followed by a fluoropolymer coating. The first layer provides electrical isolation, and the hydrophobic coating ensures low surface energy and a high contact angle. Creating these two layers is a time-consuming, multistep process that requires specialized equipment. However, manipulating reagents with high protein content inevitably leads to surface contamination known as biofouling. This unwanted deposition of proteins or other biomolecules affects the droplet transport and poses the risk of sample cross-contamination to an analyte that passes through the fouled area. Although methods for mitigating biofouling, such as using filler fluids or additives, exist [35], biofouling can not be entirely prevented. Hence, from a reliability standpoint, digital biochips are often treated as single-use disposable devices. Discarding a PCB-based digital biochip is neither cost-effective nor environmentally friendly, particularly since only the surface of the chip can potentially be contaminated.

An alternative single-step foil coating method has been demonstrated to be a feasible and inexpensive approach [5,27,36]. Foil materials such as polyethylene, Parafilm, and ethylene tetrafluoroethylene (ETFE) [27,37] can be used as a sacrificial dielectric layer that can easily be applied and replaced after use. We propose the use of plastic frames serving the purpose of foil carriers [38]. This approach effectively provides a cost-effective and sustainable digital biochip operation model; i.e., only the sacrificial coating layer is discarded rather than the complete digital biochip.

The foil-based approach fits the modularity paradigm, and it provides another reconfigurable layer to the fluidic system. Different foil materials, surface treatments, and foil frame holders can be used to adapt the digital biochip to the experimental needs.

#### 2.4.3. Top Plate

A closed digital biochip top plate is usually made of indium tin oxide (ITO)-coated glass or ITO-coated polyethylene terephthalate (PET), as illustrated in Figure 4b. Conductive layers serve as a ground electrode, and they are typically coated with a hydrophobic fluoropolymer. The already discussed biofouling issues apply to the top plates as well; hence, each needs to be replaced or cleaned if cross-contamination is a concern.

#### 2.4.4. Sensors, Actuators, and Auxiliary Devices

The variety of available sensors and actuators were mentioned in Section 1.3. Moreover, external reagents and waste reservoirs might be needed, depending on the application requirements. Pumps and connection tubing external to the digital biochip can be integrated as a part of the fluidic system. This allows the digital biochip to be used as an inline sample processor as part of a larger laboratory setup. Nevertheless, the external components interface with the supply, control, and feedback buses, thereby remaining a part of the cyber-fluidic system.

### 2.5. Instrumentation System

The instrumentation part of the cyber-fluidic architecture consists of a set of connected embedded systems. They are grouped by functionality into a modular instrumentation subsystem and a base control system, as shown in Figure 5. The base control system consists of the mainboard, power delivery, and bus interface modules. An optional display and user control modules can be added. The mainboard communicates with the virtual system over a wireless link and controls the instrumentation system. As the name implies, the power delivery module is responsible for creating and managing the power domains from a rechargeable battery pack. The bus interface module links the mainboard and the instrumentation modules, and it has a three-fold functionality. First, it provides electrical isolation between the control, feedback, and supply modules, consequently decoupling the different subsystems. Its second function is to provide the physical layer for the data links, and its third role is to deliver and monitor the power delivery to the instrumentation subsystem.

The control, feedback, and supply modules form the instrumentation subsystem, and each module is designed to address a specific digital biochip instrumentation need. For example, the minimal digital biochip instrumentation system consists of the base control system (mainboard, bus interface, and power delivery modules), an electrode driver module, and a high-voltage power supply module. The high-voltage power supply is connected to the supply bus, and it provides the required actuation voltage to the electrode driver module responsible for driving the digital biochip electrodes.

The instrumentation subsystem is assembled as a subset of a collection of existing application-specific modules, as previously illustrated in Figure 6. Additional functionality can be added on an ad hoc basis when required. For instance, interfacing to electroanalytical sensors can be added by developing a potentiostatic measurement setup and integrating it with the feedback instrumentation bus.

Virtual and physical modularity is essential for compatibility and future interoperability. Moreover, defined interfaces on the software and hardware levels are a necessity for the plug-and-play functionality. A module form factor governed by multiples of 1U ( 25 mm) provides streamlined physical organization, such as module interconnects and stacking.

### 2.6. Virtual System

The virtual system is often overlooked with the argument that the current digital biochips are still somehow simplistic devices, and their programming can mostly be managed through scripting of electrode sequences. While this approach is sufficient for a range of simple devices and applications, scripting only accounts for a humble part of the virtual infrastructure required to build fully programmable digital biochips. Furthermore, assuming that scripting electrode control sequences is an adequate programming method inevitably leads to neglecting and delaying the development of a capable synergetic virtual infrastructure for manual and automated instrumentation system control, protocol capture, translation, and execution on the digital biochip.

The manual and semi-automated system controls are essential for research and development purposes. The digital biochip processes are managed through a user interface, where the operator of the system presents the “feedback loop”—e.g., the sequence of actions and their correct completion is managed by the system operator. However, the manual control approach quickly becomes inadequate in the context of protocols running for a couple of hours, parallel handling of multiple samples, or requirements for fault-tolerant operation.

Using unstructured natural language to express a sequence of operations that build up a laboratory protocol inevitably leads to ambiguities. Consequently, domain and procedural knowledge are often required to understand, adapt, and execute the described procedure. Therefore, a methodology for capturing the protocol’s structural and behavioral semantics in an unambiguous manner is a prerequisite for the following translation and autonomous execution on a digital biochip. A structured description of laboratory protocol resembles a computer program where the reagents are depict as variables that can be operated with. This calls for developing and adopting an expressive high-level programming language that allows unambiguous protocol capturing, promoting reusability, and a paradigm shift from implementation semantics to the functional description.

Nevertheless, the concept of programming for digital biochips presents an interdisciplinary challenge where cross-domain experience is required; e.g., a user with a biochemical background is needed to deal with abstract programming concepts and modeling. Hence, the programming interface must be intuitive, convenient to learn and work with, and reasonably expressive. Accounting for the user group adoption makes the development of the protocol capture method likely the most challenging part of the virtual system.

There are a number of endeavors addressing protocol capture and programming for laboratory automation and microfluidics in general [3,24,39,40]. These languages and methods are all relatively new, and despite the significant effort, many questions remain open, such as “what makes good user experience” and “what is the essential feature set.” What stands out is the lack of standard infrastructure, including intermediate representation, execution model, and system architecture, which makes interoperability between the already demonstrated tools virtually impossible. In modern computing, this deficiency is solved by an abstract representation of a computer model known as instruction set architecture (ISA). The ISA specifies the behaviors of a low-level machine code, and it abstracts the actual hardware implementation details. This abstraction allows computer programs to be written in a different programming language, compiled for a particular ISA, and then executed without requiring comprehensive knowledge about the underlying hardware.

We believe that a μfISA is the missing link to a common cyber-fluidic architecture. Establishing an application and platform agnostic programming model will allow software and hardware module reusability similar to modern computers. We consider the platform-based modular approach discussed here as the first step towards establishing a common cyber-fluidic architecture. A high-level programming language, compiler, assembly language, and virtual machine are already under active development, and preliminary testing has already confirmed the feasibility of the modular cyber-fluidic architecture.

## 3. The Bioware Cyber-Fluidic Platform

The proposed cyber-fluidic architecture was demonstrated by building and testing a platform instance that implements the outlined concepts for modularity, reconfigurability, and programmability. Although the cyber-fluidic architecture can be realized by employing different technologies, it is important to consider that the fluidic system largely determines the functional, and consequently, the application space of a particular cyber-fluidic platform (see Figure 6). In Section 1.1 and Section 1.2, we argued in favor of digital microfluidics being a suitable liquid handling technology, able to maintain a high degree of programmability and meeting important criteria—such as integration, scalability, performance, ease of use, and cost. The instrumentation and virtual systems certainly present interesting architectural and technological challenges, but they mainly remain in the engineering domain.

The base configuration of the Bioware Cyber-Fluidic Platform (BiowareCFP) instrumentation prototype was earlier shown in Figure 1. The rest of this section discusses some of the important design aspects of the fluidic, instrumentation, and virtual systems, along with the fluidic and capabilities of the BiowareCFP.

### 3.1. The Fluidic System Implementaiton

**Figure 7:**
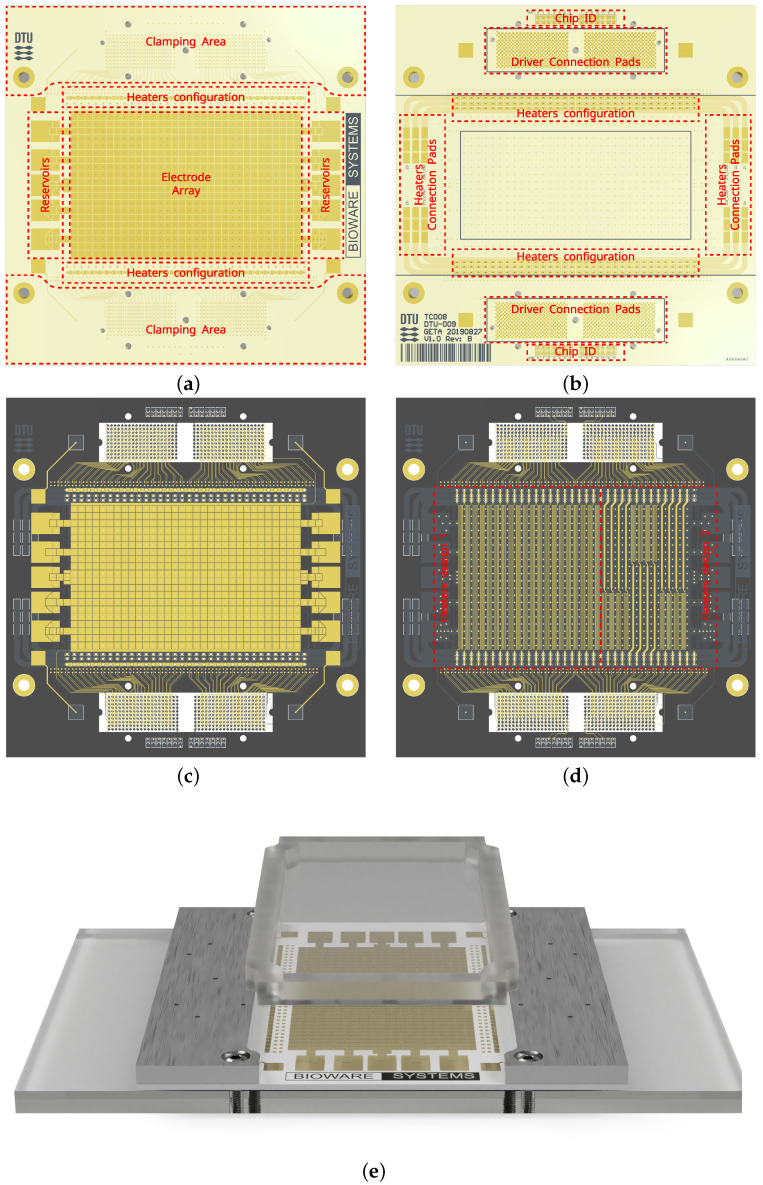
Model of the digital biochip. (**a**) Top view showing: the main actuation electrode array, the on-chip reservoirs, and the clamping area used to fix the digital biochip to the electrode drivers. (**b**) Bottom view showing the electrode driver connection pads, the heater connection pads and configuration solder bridges, and the digital biochip identification solder bridges. (**c**) The top copper layer used to fabricate the actuation electrodes. (**d**) The heaters’ copper layer is located 100μm under the top copper layer. (**e**) Digital biochip assembly and foil-frame coating.

The fluidic system is based on a PCB digital biochip, and its design is shown in Figure 7. The device’s dimensions are 100 m m× 100 mm, which correspond to 4 U × 4 U, and PCB thickness of 1.6 mm. The main electrode array consists of 640 individually addressable electrodes organized in a 32 × 20 grid, as shown in Figure 7a. The electrodes have 2 mm pitch and 89 μm spacing. Each electrode is routed to an individual connection pad located on the bottom side of the PCB, as shown in Figure 7b.

The multilayer PCB structure also allows for fabricating embedded meander structures between adjacent columns of electrodes, as shown in Figure 7c,d. Each structure forms a resistive heater that can be used to create a programmable temperature zone. Two heater designs have been implemented—the first design equaling the full 20 electrodes in height, and a second design spanning 8 electrodes, as shown in Figure 7d. The heaters can be connected in series through solder bridge connections on the top and on the bottom of the digital biochip, allowing up to eight individual heating regions spanning multiple electrode columns.

A foil-based approach was adopted for coating the electrode array. A sub- 10 μm foil was attached to a plastic frame to streamline the application process and provide a sustainable operation model generating the least amount of waste—i.e., only the foil frame assembly is exchanged. The digital biochip and foil frame assembly are shown in Figure 7e.

### 3.2. The Instrumentation System Implementation

The instrumentation system was composed of a collection of hardware modules to verify the cyber-fluidic architecture design concepts and assumptions. Eight hardware modules were developed in total; the first seven were initially intended, and the additional eighth module was developed as a result of the expansion of the application space requirements (see Figure 6). The initial set of modules consisted of the mainboard, bus interface, and power delivery modules comprising the base control system (see Figure 5); and the high-voltage power supply, electrode driver, heaters controller, and a generic prototyping module as parts of the instrumentation subsystem. The additional module is the temperature sensor interface, and it is further discussed in Section 4.

#### The Base Control System

The mainboard is the brain of the instrumentation system, and it contains various subsystems, such as communication, computation, and storage subsystems; a user interface; and interfaces for connecting external devices. The mainboard connects to the instrumentation bus interface, graphical display, user interface controls, external storage memory card, and power delivery system. The design is focused on striking a balance between being a capable, compact, and feature-rich but power-efficient embedded system that fits into the 4 U/4 U form factor.

The bus interface links the mainboard with the instrumentation modules. It implements the physical layer of the supply, control, and feedback buses and provide power to the attached modules. The three buses are electrically decoupled from each other by the use of digital isolators and separate power supply domains. The complete electrical isolation mitigates eventual cross-system disturbance and favors a fault-tolerant operation.

The power delivery module creates the multiple power domains required by the mainboard, the bus interface, and the instrumentation subsystem, and it is designed to fit the 4 U/4 U form factor. For the sake of portability, the module is designed to operate from a rechargeable battery pack, including charging and protection circuitry. The battery pack capacity can be adjusted to match the power needs of a given platform instance.

### 3.3. The Instrumentation Subsystem

Digital microfluidics requires electrode control voltages in the range of tens to hundreds of volts, depending on the dielectric properties of the coating layer [4]. A typical actuation voltage is in the range of 80–200 V, and it is sufficient for a dielectric layer thickness in the range of 5–10 μm [41]. Actuation voltages higher than 300 V are seldom reported. A high-voltage software-controlled boost power supply module was developed to meet the required voltage range. The high-voltage power supply module can produce a regulated 30–300 V output; it implements output voltage and current monitoring; and it provides adjustable output current protection.

The electrode driver module is implemented as an array of individually addressable high voltage switches providing a one-to-one mapping of driving channels to the digital biochip electrodes. This mapping scheme is the most flexible programming approach, since each control electrode has a dedicated driving channel. The electrode driver module was designed in 4 U/4 U form factor, and it has 384 driving channels that can be updated every 50 μs. The driver module also implements two capacitive sensing channels that can be used for droplet position feedback. The electrode driver module is designed to be daisy-chained, which, combined with the standard form factor, allows for easy expansion.

The heaters controller module can instrument up to three temperature regions, where each region is created from an arbitrary number of adjacent heaters connected in series. Each temperature region is controlled by a proportional–integral–derivative (PID) controller, allowing the setpoint and the thermal response of each temperature zone to be software controlled.

A generic microcontroller module and a carrier board were designed to aid the development and prototyping of the instrumentation modules. The microcontroller module was used as a base for every instrumentation module, embracing design reusability on hardware and software levels.

### 3.4. The Virtual System

The platform-based design allows system development to be carried out simultaneously from top-down and bottom-up system perspectives. The proposed cyber-fluidic architecture holds the potential to combine the high-level synthesis tools [42] with the envisioned universal μfISA and a low-level digital biochip implementation [38]. Indeed, the instrumentation system presented here is where the μfISA meets with the high-level tools. The conceptualized μfISA (see Section 2.6) is under active development. It includes a software framework for protocol capture, compilation, domain-specific assembly language, and a virtual execution environment. Currently, the μfISA is undergoing testing and integration with the presented cyber-fluidic platform.

## 4. Results and Discussion

Understanding and addressing the intrinsic relationships spanning across the system’s domains is essential for overall cyber-fluidic architecture. Nevertheless, foreseeing all possible benefits and shortcomings of a given approach in the context of a full system is virtually impossible, especially considering the constant technological developments. To deal with the complexity and uncertainty, we devised a modular platform-based cyber-fluidic architecture. The separation of the fluidic, instrumentation, and control concerns, plus the modularization within each system, create an evolvable platform framework that can be modified and expanded to meet virtually any emerging need.

**Figure 8:**
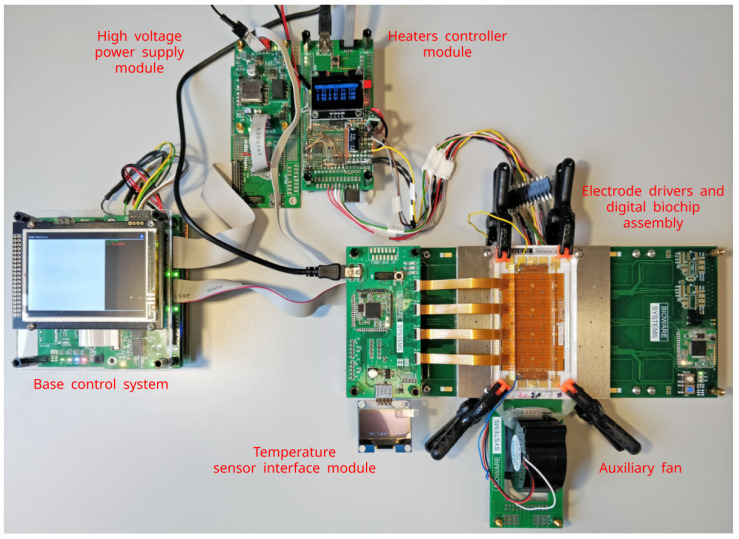
The instrumentation system for calibrating three temperature zones for space domain PCR.

The baseline functionality of BiowareCFP was tested by performing single and parallel droplet manipulations in an open and closed digital biochip (see Supplementary Videos S1–S4). While the minimal BiowareCFP instance provides the means for EWOD droplet actuation, adding the heaters controller module (see Figure 8) allows for creating three individual temperature zones for performing space domain polymerase chain reaction (PCR) on the digital biochip. Multiple tests were carried out in order to make BiowareCFP ready for space domain PCR. An unforeseen overshoot of the actual temperature (compared to intended) was revealed when the embedded heaters were used in a closed digital biochip configuration. The problem was narrowed down to the thermal response of the closed digital biochip, and to be solved, it required developing a calibration technique for multipoint temperature measurement that fit into the 300 μm between the bottom and top plates of the digital biochip.

**Figure 9:**
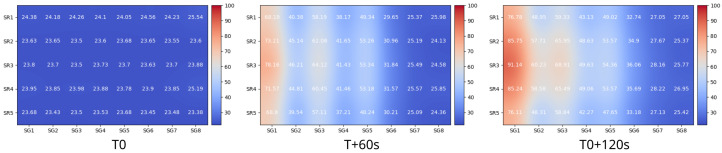
Temperature regions characterization for space-domain PCR. Each heatmap shows the measured temperature profile of the full electrode array at 0, 60, and 120 seconds after activating the three individual temperature zones. Starting at room temperature (T0), the three temperature zones reached their set points at 92 ${}^{\circ }C$, 68 ${}^{\circ }C$, and 54 ${}^{\circ }C$ in 120 s, as shown in T0 + 120s.

The temperature sensor array and a corresponding instrumentation module were developed to assist with profiling and calibrating the temperature response of the digital biochip. The module allowed for real-time capturing of 40 temperature points distributed over the electrode array. The captured data were used for fine-tuning the thermal performance of the heating zones in a realistic operational environment. A heatmap sequence visualization of the generated three temperature zones in a closed digital biochip is shown in Figure 9. The heatmap sequence shows the temperature profile developed over two minutes, starting with room temperature and reaching the 92 ${}^{\circ }C$, 68 ${}^{\circ }C$, and 54 ${}^{\circ }C$ required for the space-domain PCR.

The development and straightforward integration of the temperature sensor array and instrumentation module with the rest of the BiowareCFP serves as a clear example of the advantages of the modular platform-based approach. The modularity of the fluidic system, e.g., the removable top plate, allowed the temperature sensor array to be installed between the digital biochip and the top plate, and the removable foil frame could be replaced after calibrating the temperature response of the system. The modular instrumentation subsystem provided a standard interface to the rest of the BiowareCFP. Moreover, the addition of the heaters controller and the temperature sensor array and interface modules can be used as practical examples of the extensibility and reconfigurability of the proposed cyber-fluidic architecture. This can be seen in Figure 8, where the minimal BiowareCFP instance, previously shown in Figure 1, had been extended with the discussed additional modules to make the BiowareCFP capable of running PCR.

Preliminary results from the implementation and testing of the envisioned μfISA and the corresponding virtual execution environment show excellent programmability potential. Droplet routing with real-time flow-control has already been tested. This allows for conditional execution of laboratory protocols where sensory input from the fluidic systems is fed back to the virtual execution environment. The software toolchain is under active development, and its progress is considered one of the crucial next steps in the development of the envisioned full-stack cyber-fluidic architecture.

There are still various engineering challenges to be addressed, such as integrating biosensors and designing the corresponding instrumentation modules. However, based on the developed framework and the high degree of modularity, we argue that the BiowareCFP provides an application-agnostic approach for developing cyber-fluidic system. Preliminary results on performing space domain PCR as a part of cell cloning and magnetic beads-based ELISA can be found in [38]. The results will be published when the applications have been thoroughly investigated and reproduced.

## 5. Conclusions

In addition to the technological challenges, substantial performance, programmability, reliability, and socioeconomic roadblocks need to be overcome before the cyber-fluidic systems realize their full potential as capable fluidic handling technology. We argue about the importance of standardization, unification, and reusability, which should significantly accelerate the technology transfer process. Moreover, we advocate for a strong interdisciplinary approach to digital microfluidics in order to raise awareness and emphasize the need for a holistic approach that has the merits to establish the envisioned universal microfluidic framework.

We introduced the term cyber-fluidic systems and established the groundwork for implementing the envisioned framework based on digital microfluidics. Although we realize that the proposed architecture might not be the panacea for solving all microfluidics challenges and likely has limitations, it is a step towards more capable and integrated cyber-fluidic systems. We consider the implementation of the BiowareCFP to blur the boundaries between the software and fluidic domains and start the shift towards Bioware systems—a concept signifying the seamless integration between the cyber-fluidic system and biology. A particularly relevant class of applications comes from the synthetic biology field, where the BiowareCFP provides the means to create or modify biological parts, reprogram living cells, and to be used for biological computing. It has been an exciting couple of decades for the microfluidics field, and we believe the highly anticipated “killer application(s)” and consequent commercialization are on their way.

## Supplementary Materials

The following supporting information can be downloaded at: https://www.mdpi.com/article/10.3390/mi13020249/s1, Video S1: Open Biochip Moving Droplets, Video S2: Closed Biochip Droplet Dispensing, Video S3: Closed Biochip Droplet Splitting, Video S4: Closed Biochip Droplet Mixing.
